# Reduce, Reuse and Recycle in Protein Chromatography: Development of an Affinity Adsorbent from Waste Paper and Its Application for the Purification of Proteases from Fish By-Products

**DOI:** 10.3390/biom10060822

**Published:** 2020-05-27

**Authors:** Georgios E. Premetis, Nikolaos E. Labrou

**Affiliations:** Laboratory of Enzyme Technology, Department of Biotechnology, School of Applied Biology and Biotechnology, Agricultural University of Athens, 75 Iera Odos Street, GR-11855 Athens, Greece; giorgos.prem@gmail.com

**Keywords:** affinity chromatography, cellulose adsorbents, Cibacron Blue 3GA, fish by-products, proteases

## Abstract

In the present study, we report the development of a cellulose-based affinity adsorbent and its application for the purification of proteases from fish by-products. The affinity adsorbent was synthesized using cellulose microfibers as the matrix, isolated from recycled newspapers using the acid precipitation method. As an affinity ligand, the triazine dye Cibacron Blue 3GA (CB3GA) was used and immobilized directly onto the cellulose microfibers. Absorption equilibrium studies and frontal affinity chromatography were employed to evaluate the chromatographic performance of the adsorbent using as model proteins bovine serum albumin (BSA) and lysozyme (LYS). Absorption equilibrium studies suggest that the adsorption of both proteins obeys the Langmuir isotherm model. The kinetics of adsorption obey the pseudo-second-order model. The affinity adsorbent was applied for the development of a purification procedure for proteases from *Sparus aurata* by-products (stomach and pancreas). A single-step purification protocol for trypsin and chymotrypsin was developed and optimized. The protocol afforded enzymes with high yields suitable for technical and industrial purposes.

## 1. Introduction

The development of chromatographic materials for the separation and purification of proteins has been an essential tool for research and development in biotechnology [1,2,3]. The growing of biotechnology, to a certain extent, relays on the development of protein purification methods and protocols [4,5,6]. However, protein purification cost remains high, and constitutes a substantial proportion of the overall production cost [2,3]. Purification methods that are designed based on specific, effective and robust materials are expected to guide the future of the protein purification area [6,7]. Affinity chromatography is the most specialized method for the effective purification of proteins, compared to other separation methods [2,3,4,8,9,10,11,12,13]. It offers high selectivity, resolution, and capacity in most protein purification procedures. Affinity chromatography has the advantage of exploiting a protein’s structure or function (molecular recognition) as it is based on the specific and reversible interaction between the target protein with an immobilized ligand [2,3,4,8,9]. The interaction between the target protein and the immobilized ligand is the result of various molecular interactions involved, namely, electrostatic, hydrogen bonding, hydrophobic and van der Waals interactions [14].

The immobilized ligand is the key factor that affects the effectiveness of any affinity chromatographic method, since it provides the selectivity with the target protein [1]. However, the matrix itself is an additional parameter that determines the performance of the affinity chromatography as it affects the specificity of interaction, the capacity for the target protein as well as the stability of the adsorbent [15,16]. Examples of frequently used support matrices are agarose, cellulose, dextran, silica, glass and polyacrylamide derivatives. Cellulose exhibits sufficient chemical, biological and mechanical stability to justify its use as chromatographic material [17].

In the present study, we employed the triazine dye Cibacron Blue 3GA (CB3GA) as an immobilized ligand. Triazine dyes display several advantages compared to specific biological ligands due to their low cost, resistance to biological and chemical degradation and high protein binding capacity [10,11,12,13,18,19,20,21,22]. In addition, the presence of the triazine ring allows their direct immobilization to the matrix (e.g., Sepharose or cellulose) through a nucleophilic substitution reaction [15,16].

Fish and other marine organisms are promising sources of unique enzymes with biotechnological potentials [23]. Particularly, fish by-products are a rich source of proteases and other biotechnological enzymes, which have high commercial value [24]. Proteases are the most widely used enzymes, covering an array of applications, from the food industry to cosmetics and medicine [25]. Trypsin and chymotrypsin belong to the serine proteases family of enzymes, which are commonly found in pancreatic extract and stomach [25,26].

“Waste hierarchy” is the order of priority of actions to be taken to reduce the amount of waste generated, and to improve overall waste management processes and programs. The waste hierarchy consists of 3R’s: Reduce, Reuse and Recycle. The 3R’s of waste management is the guidance suggested for creating a sustainable life [26]. Along these aspects, the present work was undertaken in order to design a strategy for developing affinity chromatographic materials and protocols for more sustainable bioeconomy and bio-based products.

## 2. Materials and Methods

### 2.1. Materials

Sodium phosphate, sodium chloride, triazine dye Cibacron Blue 3GA and the protease substrates N-benzoyl-L-tyrosine ethyl ester (BTEE) and N^α^-benzoyl-L-arginine ethyl ester (BAEE) were obtained from Sigma-Aldrich (Darmstadt, Germany). Potassium thiocyanate (KSCN) was obtained from Carlo Erba (Chaussée du Vexin, France). Hydrogen chloride (HCl) was obtained from Scharlab (Barcelona, Spain). All other chemicals were of analytical grade and purchased from Merck (Darmstadt, Germany). The structures were created using ChemDraw Ultra 12.0 (CambridgeSoft, Cambridge, MA, USA, www.cambridgesoft.com).

### 2.2. Methods

#### 2.2.1. Extraction and Characterization of Cellulose Microfibers from Waste Paper

Cellulose from waste newspapers was extracted as described by Takagi et al., 2013 [27] with some minor modifications. Before the alkaline treatment, newspapers were boiled until a pulp was formed. The alkaline treatment was conducted for 3 h at 70 °C, followed by several washing cycles until the pH of the washings reached a neutral value. The bleaching treatment for the removal of the colored substances and the remained lignin was carried out using an aqueous solution of commercial sodium hypochlorite (2% *w*/*v*). This treatment was continued for 12–14 h at room temperature, following by several washes for the complete removal of colored substances. Then, the cellulose pellet was subjected to an acid hydrolysis by an aqueous solution of hydrogen chloride (64% *v*/*v*) for 1 h at 35 °C. The cellulose microfibers were assessed and visual inspected using the optical microscope OLYMPUS U-CMAD3 (Olympus Europa SE & Co., Kg, Hamburg, Germany) using the lens OLYMPUS Dx4, Dx10 and Dx20 and the images were processed using Image J. Each sample was suspended in distilled water before observations.

#### 2.2.2. Immobilization of Cibacron Blue 3GA on Cellulose Microfibers

The immobilization of CB3GA on cellulose microfibers was accomplished as described previously [15,16], with minor modifications. The nucleophilic substitution reaction was carried out at 60 °C under stirring for 7.5 h, using 28 mg CB3GA per g of cellulose microfibers. CB3GA was immobilized on cellulose microfiber before and after its acid hydrolysis step. The resulting adsorbents, denoted as CB3GA-Cellulose-1 and CB3GA-Cellulose-2, respectively, were used. The immobilized dye concentration (μmol dye/g of dry adsorbent) was determined as described by Chronopoulou and Labrou, 2014 [14], using as molar extinction coefficient 5.4 L mmol^−1^ cm^−1^.

#### 2.2.3. Determination of Protein Dynamic Capacity for the Affinity Adsorbents

Frontal affinity chromatography was used for the determination of the adsorbents’ (CB3GA-Cellulose-1 and CB3GA-Cellulose-2) capacity for the two standard proteins: bovine serum albumin (BSA) and lysozyme (LYS). All procedures were performed at 4 °C. Affinity adsorbents (0.5 mL moist wet gel, 51 mg dry weight) were washed with 10 mL double distilled water and equilibrated with 10 mL potassium phosphate buffer (10 mM, pH 7.0). Protein (BSA or LYS) in 10 mM potassium phosphate buffer pH 6.0 was loaded on the adsorbent (10 mg total protein) and effluents were collected in 2 mL fractions. Bound BSA or LYS were eluted with 3M sodium chloride (pH 7.0). Total protein in the collected fractions was determined by Bradford assay [28].

#### 2.2.4. Absorption Equilibrium Studies

Adsorption equilibrium studies were carried out as described previously [24,25,26,27], with minor modifications: in a total volume of 1 mL (10 mM potassium phosphate buffer, pH 7.0), varying amounts of protein (BSA or LYS; 20–100 μg) previously dissolved in potassium phosphate buffer (10 mM, pH 7.0), were mixed with 20 mg of the affinity adsorbent CB3GA-Cellulose-2. The suspensions were placed in a rotary shaker for 75 min at 4 °C in order for the system to reach equilibrium. The mixture was then centrifuged (13,000× *g*, 2 min) and the amount of unbound protein in the supernatant was determined by Bradford assay [28].

The equilibrium can be represented by a second-order reversible interaction (Equation (1)):

(1) $$E+D\overset{K_{1}}{\underset{K_{2}}{⇄}}ED$$

where *E* is the enzyme in solution, *D* is the ligand (adsorption site), *ED* is the enzyme-ligand reversible complex, K_1_ and K_2_ are the forward and reverse rate constants, respectively. The ratio K_1_/K_2_ equals the equilibrium dissociation constant (*K_D_*) of the enzyme-ligand complex. The Langmuir isotherm (Equation (2)) has been widely used to describe protein adsorption onto a wide range of affinity adsorbents [29,30,31,32]:

(2) $$q*=\frac{c*q_{\max}}{K_{D}+c*}$$

where *q* and *c* are the equilibrium concentrations of the adsorbed protein and protein in the solution, respectively, *q*_max_ is the maximum adsorption capacity and *K_D_* is the equilibrium dissociation constant. Different values of c* and q* parameters are taken in order to fit the data using the Langmuir isotherm. Transforming the equation of Langmuir isotherm into double reciprocal, the following equation can be obtained:

(3) $$\frac{c*}{q*}=\frac{K_{D}}{q_{\max}}+\frac{c*}{q_{\max}}$$

#### 2.2.5. Absorption Kinetic Studies

Adsorption kinetics studies were carried out as previously described [29,30,32], with minor modifications: in a total volume of 4 mL (10 mM potassium phosphate buffer pH 7.0, protein (BSA or LYS, 5 mg total protein), in 10 mM potassium phosphate buffer pH 7.0, was mixed with the CB3GA-Cellulose-2 affinity adsorbent (100 mg moist wet gel, 9.3 mg dry mass). The suspension was shaken at 4 °C. Adsorption velocity was monitored by periodically removing samples from the suspensions, which were subsequently centrifuged (10,000× *g*, 30 s), and the amount of total protein concentration in the supernatant was determined by the Bradford method [28]. In order to evaluate the mechanism that controls the adsorption process, three adsorption models were evaluated: the pseudo first-order, the pseudo-second-order and the pore diffusivity model [33,34,35,36]. The best-fit model was selected based on the linear regression correlation coefficient (R^2^). The pseudo-second-order model showed the best correlation with the experimental data compared to the other two models. If the rate of adsorption has a second-order mechanism, the pseudo-second-order chemisorption kinetic rate equation is expressed by Equation (4) [33,34,36]:

(4) $$\frac{dq_{t}}{dt}=k_{2}{\left(q_{e}-q_{t}\right)}^{2}$$

where: *q_e_* and *q_t_* are the adsorption capacities at equilibrium and at time *t* (min), respectively, and *k*_2_ (mg protein mg^−1^ min^−1^) is the rate constant for pseudo second-order adsorption. The linear form of the pseudo-second-order equation is given by Equation (5):

(5) $$\frac{t}{q_{\left(t\right)}}=\frac{1}{k_{2}q_{e}^{2}}+\frac{1}{q_{e}}t$$

The product *k*_2_*q_e_*^2^, also represented by h (mg mg^−1^ min^−1^), corresponds to the initial adsorption velocity. The plot of *t*/*q_t_* vs. *t* is linear [33] and therefore the constants *q_e_* and *k*_2_ can be determined from the slope and intercept of the straight line [34,35,36].

#### 2.2.6. Enzyme Assay

Determination of total protease activity was based on the protocol of Côlho et al., 2016 [37], using azocasein as substrate. One unit of protease activity is defined as the amount of the enzyme that produces a 0.01 increase in absorbance at 440 nm. Determination of chymotrypsin activity was carried out using N-benzoyl-L-tyrosine ethyl ester (BTEE) as the substrate [38]. Determination of trypsin activity was achieved using N^α^-benzoyl-L-arginine ethyl ester (BAEE) as the substrate [38]. All measurements were performed in triplicate.

#### 2.2.7. Extraction of Proteases from *Sparus aurata* Stomach and Pancreas

Stomach or pancreas (1 g fresh weight) were cut into small pieces and suspended in 3 mL of 10 mM potassium phosphate buffer, pH 7. The mixture was subsequently centrifuged at 10,000× *g* for 20 min at 4 °C. The supernatant was collected for further use.

#### 2.2.8. Affinity Chromatography of Proteases from *Sparus aurata* Stomach and Pancreas

Crude extract from *Sparus aurata* stomach or pancreas was loaded on the affinity adsorbent CB3GA-Cellulose-2 (0.5 mL moist adsorbent). The column was washed with 10 mM potassium phosphate buffer, pH 6.5, prior to elution with 3 M KCl, dissolved in 10 mM potassium phosphate buffer, pH 6.5. The flow-through and eluted fractions were collected and the total protein was determined by the Bradford method [28]. The column was regenerated with 3M potassium thiocyanate.

## 3. Results and Discussion

### 3.1. Extraction and Characterization of Cellulose Microfibers from Waste Paper (Newspaper)

**Figure 1:**
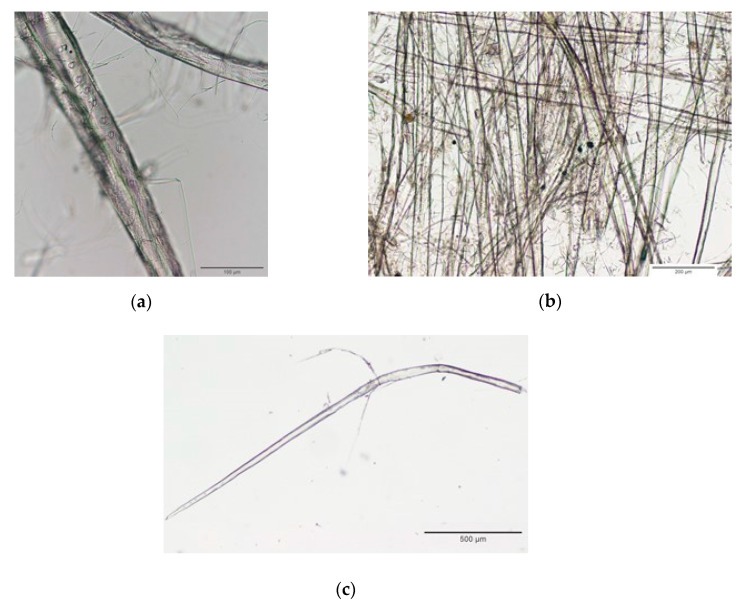
Optical microscopy of cellulose microfibers. The cellulose microfibers were assessed and visual inspected using the optical microscope OLYMPUS U-CMAD3 using the lens OLYMPUS Dx4, Dx10 and Dx20. Scale bar, 100 (**a**), 200 (**b**) and 500 μm (**c**). Images were processed using Image J.

Optical microscope was used to determine the fiber dimensions as well as to visualize the fracture surface of the cellulose microfibers. Visual inspection of the extracted cellulose microfibers using a microscope (Figure 1) indicated that their morphology exhibited a rod-like microstructure, with some individual cellulose microfibers arranged longitudinally, presumably due to hydrogen bonding network among macro-scale cellulose microfibers [39]. The cellulose matrix is shown in Figure 1A. Microfibers appear to be embedded in the matrix, organized in bundles and their size varies between 100 and 1500 μm, with some of the fibers being arranged longitudinally and attached to each other by hydrogen bonds This morphology and fibers length is in agreement with previously published works [27,40,41,42]. The cellulose microfibers isolated from waste newspaper appeared to be less uniform, which can be attributed to the possible uncontrolled cleavage of cellulose chains during acid hydrolysis [27]. 

### 3.2. Synthesis of the Affinity Adsorbent

**Figure 2:**
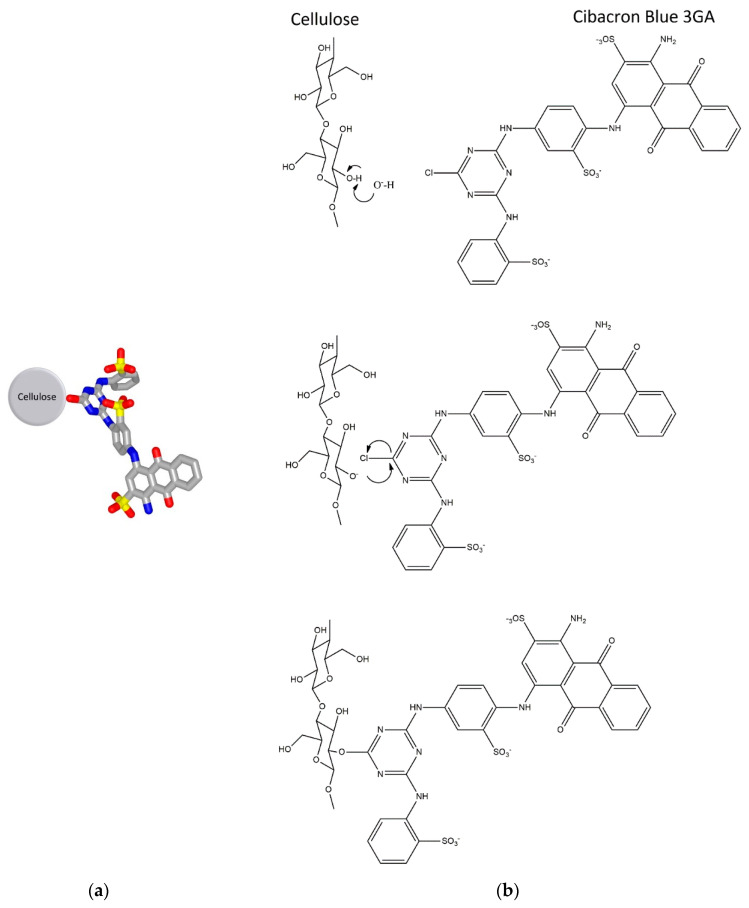
(**a**) The putative structure of the affinity adsorbent. The immobilized ligand is the triazine dye CB3GA. (**b**) The synthetic route for the synthesis of the affinity adsorbent. The structures were created by ChemDraw Ultra 12.0.

The triazine dye, CB3GA, is a well-established ligand in affinity chromatography [2,3,8,9,41]. The presence of hydrophobic, ionic and aromatic moieties in CB3GA give rise to the formation of mixed type interactions with proteins such as electrostatic, hydrophobic, hydrogen bonding interaction [11,12,13,20,21,22,43,44]. The presence of the chlorotriazine ring in CB3GA allows its direct immobilization onto the matrix. This is achieved through a nucleophilic substitution reaction of the electrophile chloride of the chlorotriazine group by the hydroxyl groups of the cellulose microfibers (Figure 2).

The concentration of the immobilized dye was determined 3.55 and 3.99 μmol dye/g dry adsorbent for the CB3GA-Cellulose-1 and CB3GA-Cellulose-2, respectively. The concentration of the immobilized dye is a crucial parameter in dye-ligand affinity chromatography, as it defines the capacity and specificity of the adsorbent for the target protein [43,45]. In particular, high concentration of the dye-ligand leads to lower specificity and capacity, since excessive levels of dye promote nonspecific protein binding. In addition, it can restrict the ability of the target protein to form specific complex with the immobilized dye as a consequence of steric effect [15,45]. Moreover, low level of immobilized ligand leads to lower binding capacity for the target protein. An optimum ligand concentration, which allows, on one hand, specific protein binding and on the other hand, high capacity lies between 3.0 ± 1.0 μmol dye/g dry adsorbent [2,15,46,47,48].

**Figure 3:**
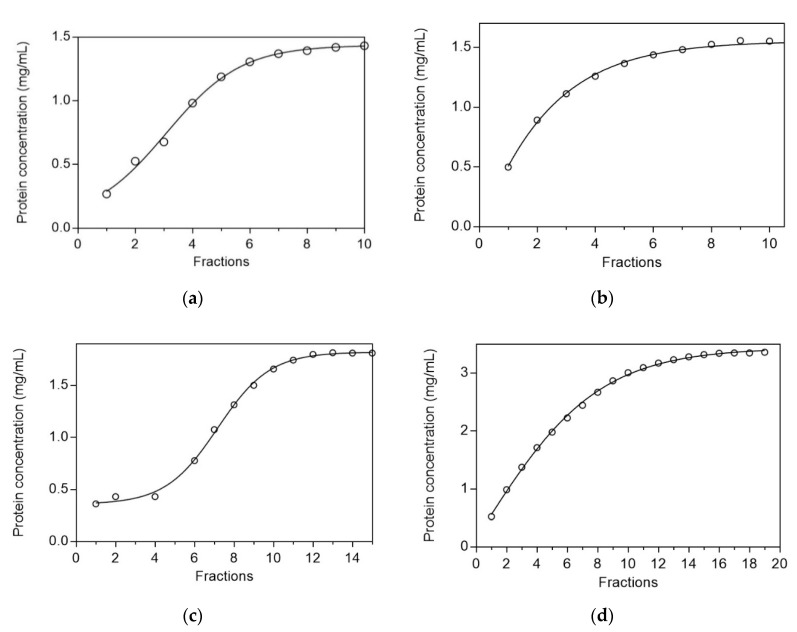
Dynamic capacity of bovine serum albumin (BSA) and lysozyme (LYS) on the affinity adsorbents CB3GA-Cellulose-1 and CB3GA-Cellulose-2. (**a**) Dynamic capacity determination of BSA for CB3GA-Cellulose-1. (**b**) Dynamic absorption capacity determination of LYS for CB3GA-Cellulose-1. (**c**) Dynamic capacity determination of BSA for CB3GA-Cellulose-2. (**d**) Dynamic absorption capacity determination of LYS for CB3GA-Cellulose-2. Eluted fractions (2 mL) were collected and total protein was determined by the Bradford method [28].

Frontal analysis was employed to evaluate the dynamic capacity and chromatographic performance of the CB3GA-Cellulose-1 and CB3GA-Cellulose-2 affinity adsorbents, using two standard proteins (BSA and LYS) and the results are shown in Figure 3. BSA and LYS are two well-studied proteins, which are frequently used as models in protein chromatography [13,20,21]. They differ in molecular weight and isoelectric point (pI) and thus, provide useful conclusions concerning the chromatographic behavior of many adsorbents. As shown in Figure 3, CB3GA-Cellulose-2 exhibited higher dynamic capacity for both proteins, compared to CB3GA-Cellulose-1. In particular, the adsorption dynamic capacity of BSA was 1.4 and 1.8 mg/mL for CB3GA-Cellulose-1 and CB3GA-Cellulose-2, respectively, however the capacity for LYS was 1.5 and 3.3 mg/mL for CB3GA-Cellulose-1 and CB3GA-Cellulose-2, respectively. In addition, the gradient of the curve in Figure 3 gives an indication about the specificity of binding [49]. The steeper curves obtained for CB3GA-Cellulose 2, compared to CB3GA-Cellulose 1, suggests more specific binding of BSA and LYS to CB3GA-Cellulose-2, compared to CB3GA-Cellulose-1. Thus, CB3GA-Cellulose-2, appears to display better chromatographic performance and therefore, it was selected for further study.

### 3.3. Adsorption Equilibrium and Kinetics Studies

**Figure 4:**
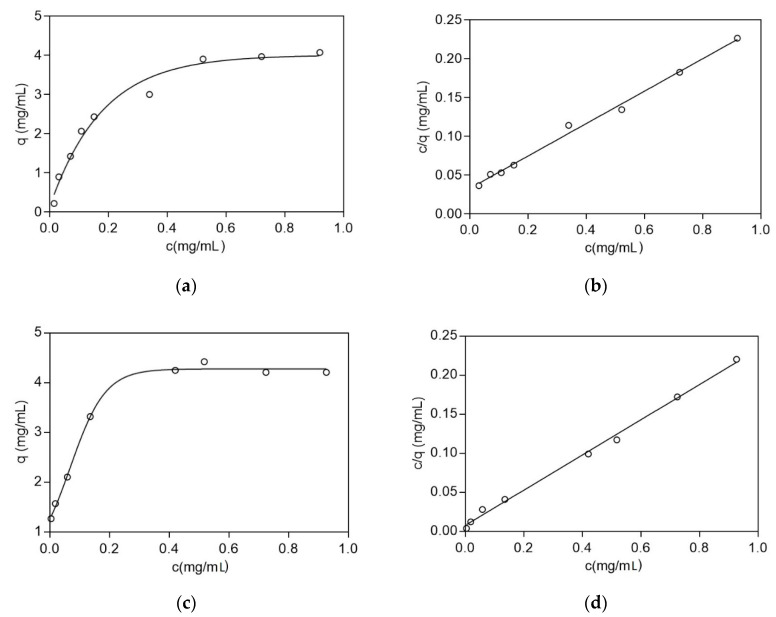
Adsorption of BSA and LYS on CB3GA-Cellulose-2 adsorbent. The plots depict the equilibrium in liquid phase protein concentration (c, mg/mL) vs. the equilibrium in solid-phase protein concentration (q, mg/mL adsorbent). (**a**) Langmuir isotherm of the absorption of BSA. (**b**) Linear regression of the Langmuir equation for BSA. (**c**) Langmuir isotherm of the absorption of LYS. (**d**) Linear regression of the Langmuir equation for LYS.

The interaction of the adsorbent CB3GA-Cellulose-2 with BSA and LYS was evaluated employing adsorption equilibrium studies [28,29,30,31]. Adsorption equilibrium studies are usually used to characterize the dynamic equilibrium between the concentration of the protein that is in the solution, and the protein that is adsorbed to the matrix [28,29]. When the protein interacts with the ligand an equilibrium is reached between the protein in the solution and the protein bound to the adsorbent. The Langmuir isotherm has been widely employed for the study of protein adsorption onto a wide range of affinity adsorbents [30,31]. As shown in Figure 4, the binding of the protein increases sharply at low protein concentrations, however, at higher concentrations is reaching a limiting value (plateau), suggesting that the adsorbent is “saturated” by protein molecules. The equilibrium adsorption data, for the adsorption of BSA and LYS on the CB3GA-Cellulose-2 affinity adsorbent, were well fitted by a Langmuir isotherm (R^2^ = 0.9943 for BSA and R^2^ = 0.9964 for LYS). Langmuir parameters for the interaction of BSA and LYS with the CB3GA-Cellulose affinity adsorbent are summarized in Table 1.

**Figure 5:**
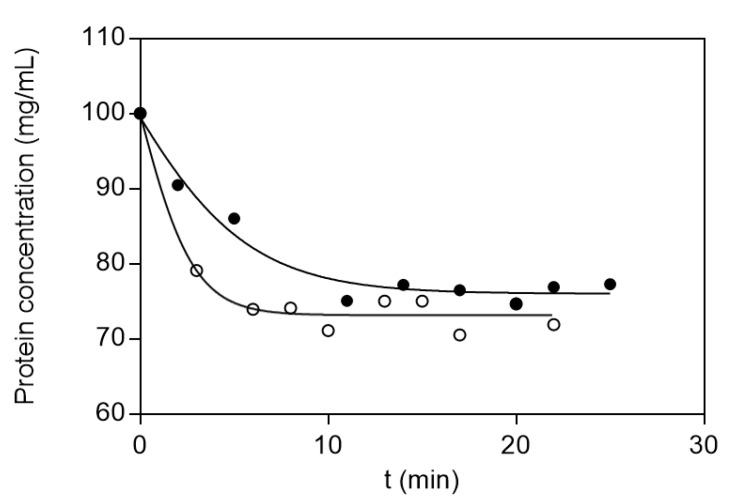
Equilibrium adsorption kinetics of LYS (white plots) and BSA (black plots) on CB3GA-Cellulose-2 adsorbent in batch system at 4 °C.

**Figure 6:**
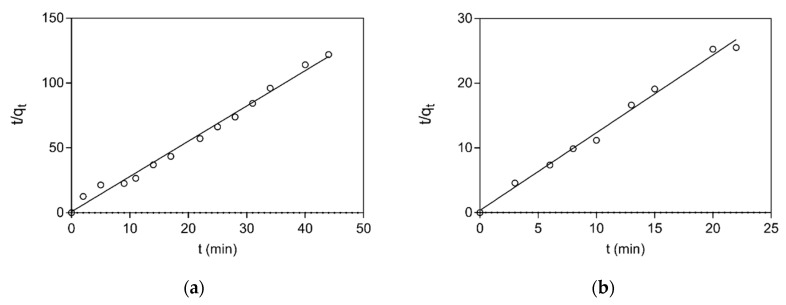
Pseudo-second order adsorption kinetics of BSA (**a**) and LYS (**b**) onto CB3GA-Cellulose-2 adsorbent.

In addition, to further investigate the rate and the mechanism of adsorption, kinetics studies were employed. Figure 5 depicts the adsorption of BSA and LYS to CB3GA-Cellulose-2 adsorbent. The results showed that the adsorption has sufficiently rapid association kinetics and the equilibrium is restored after about 5 min for LYS and after about 10 min for BSA. Several models have been published in the literature to describe kinetic models for adsorption, with the pseudo-first-order, the pseudo-second-order and the pore diffusivity model being the most frequently employed [33,34,35,50,51]. Thus, these three kinetic models were applied for determining the rate and mechanism of adsorption for LYS and BSA onto the adsorbent. The correlation coefficient R^2^ was used for assessing which model is more appropriate for describing the adsorption. The pseudo-second-order model showed the best correlation (R^2^ = 0.990 for BSA; R^2^ = 0.992 for LYS) with the experimental data, for both proteins, compared to the other two models (e.g., pseudo first-order and pore diffusivity model). The adsorption kinetics of BSA and LYS onto CB3GA-Cellulose-2 adsorbent is shown in Figure 6. The results indicate that the overall rate of the BSA and LYS adsorption process is most likely to be controlled by the chemisorption process [32,33,34,35]. The best fit values of h, *q_e_* and *k*_2_ for the pseudo-second-order models are shown in Table 2.

### 3.4. Development and Optimization of the Purification Protocol of Proteases from Sparus aurata on the Adsorbent CB3GA-Cellulose-2

A detailed study was carried out for developing an optimized protocol for the purification of proteases from stomach and pancreatic extract of *Sparus aurata* on CB3GA-Cellulose-2 adsorbent. The effect of pH on adsorbent’s capacity, purifying ability and yield towards the target enzyme activity, was investigated (Table 3). It is well established that the pH of the binding and elution buffer is one of the most crucial parameters in dye-ligand affinity chromatography as it can affect dramatically the chromatographic behavior, such as affinity, selectivity and recovery of the target protein [29,30,41]. The results showed that at acidic pH values the binding capacity is enhanced, compared to that at neutral pH. Acidic conditions are more likely to promote stronger interaction between the negatively charged CB3GA with proteins. Different pH values were also assessed in the elution buffers in terms of enzyme yield and purity (Table 3). The optimal chosen conditions, which demonstrate the highest levels of capacity, recovery and purity for the target proteases were: 10 mM potassium phosphate buffer, pH 5.5 for the equilibration buffer, 10 mM potassium phosphate, and pH 6.5 (contained 3M sodium chloride) for the elution buffer. The results of a typical purification are summarized in Table 4. The protocol afforded trypsin and chymotrypsin with high yield suitable for technical and industrial purposes.

## 4. Conclusions

The present study demonstrated that wastepaper such as newspaper can serve as a source of cellulose microfibers, suitable for the synthesis of affinity adsorbents. The advantages of such affinity adsorbents may be summarized as follows: (i) the affinity ligand CB3GA is stable and resistant to chemical and biological degradation; (ii) the method for the synthesis of such affinity adsorbents is easy and it is based on low-cost materials and reagents; (iii) it is based on reused starting material and therefore offers positive environmental impact; (iv) the binding (10 mM potassium phosphate buffer, pH 5.5) and elution (10 mM potassium phosphate buffer pH 6.5) conditions for the target protein are mild and therefore protein denaturation phenomena are minimized. For example, the eluted enzyme retained its activity for several days when stored at 4 °C; (v) the proposed purification protocol could easily be adapted at industrial scale and offers the scalability and economy required to meet the anticipated demand for such products at affordable market price.

## Figures and Tables

**Table 1:** Langmuir parameters for the interaction of BSA and LYS with the CB3GA-Cellulose-2 affinity adsorbent.

Protein	Dissociation Constants (*K_D_*) (mg/mL)	Maximum Adsorption Capacity (*q*_max_) (mg/mL)
BSA	0.16	4.78
LYS	0.03	4.43

**Table 2:** Pseudo second-order adsorption rate constants for the adsorption of LYS and BSA on CB3GA-Cellulose-2 affinity adsorbent.

Protein	*k*_2_ (mg Absorbent/mg Protein min)	*q_e_* (mg Protein/mg)	h (mg Protein/mg min)	R^2^
BSA	0.76	0.39	5.64	0.990
LYS	0.35	0.90	0.51	0.992

**Table 3:** The effect of pH of the equilibration buffer on the chromatographic behavior (capacity, purifying ability and yield) of total protease activity on CB3GA-Cellulose-2 affinity adsorbent.

pH of the Equilibration Buffer (Binding)	pH of the Equilibration Buffer (Elution)	Purifying Ability (Purification Fold)	Capacity (%) *	Yield (%)
7.0	7.0	9	25	8.5
6.5	6.5	11	25	46.7
5.5	6.5	47.8	100	167.8

* Capacity was expressed as % of the best condition that corresponds to pH 5.5.

**Table 4:** Purification protocol for proteases from *Sparus aurata* (stomach and pancreas) on CB3GA-Cellulose 2 adsorbent.

Step	Activity (Units)	Protein (mg)	Specific Activity (U/mg)	Purification (fold)	Yield (%) *
**Protease from stomach extract**					
Crude extract	7.07	10.51	0.67	1	100
Affinity chromatography	11.87	0.37	32.08	47.88	167.89
**Protease from pancreatic extract**					
Crude extract	7.87	1.81	4.35	1	100
Affinity chromatography	8.92	0.06	148.67	34.18	113.34
**Trypsin**					
Crude extract	960.32	1.81	530.56	1	100
Affinity chromatography	471.06	0.06	7851.00	14.80	49.05
**Chymotrypsin**					
Crude extract	4.56	1.81	2.52	1	100
Affinity chromatography	0.86	0.06	14.33	5.69	18.86

* The yield (%) was calculated as the ratio: (eluted enzyme units/bound enzyme units) × 100.
